# Capillary Zone Electrophoresis for the Analysis of Phenolic Preservatives in Plasma and Insulin Formulations Using Salting‐Out Assisted Liquid–Liquid Extraction

**DOI:** 10.1002/elps.70083

**Published:** 2026-03-11

**Authors:** Meriem Dadouch, Yoann Ladner, Catherine Perrin

**Affiliations:** ^1^ Département de Pharmacie, Faculté de Médecine Taleb Mourad Université Djillali Liabes de Sidi Bel Abbès Sidi Bel Abbès Algérie; ^2^ Laboratoire de Chimie Physique des Macromolécules et des Interfaces Biologiques (LCPMIB) Université de Mascara Mascara Algérie; ^3^ Institut des Biomolécules Max Mousseron (IBMM) UMR 5247‐CNRS‐UM‐ENSCM) Université de Montpellier Montpellier France

**Keywords:** capillary zone electrophoresis, human plasma, insulin formulations, phenolic preservatives, salting‐out assisted liquid–liquid extraction

## Abstract

Phenolic preservatives are critical excipients in biopharmaceutical formulations such as insulin and its analogs. The determination of these analytes in insulin formulations and human plasma is crucial for various applications. This study reports the first use of a method combining salting‐out assisted liquid–liquid extraction (SALLE) with capillary zone electrophoresis (CZE). The CZE‐based separation of phenolic preservatives remains particularly challenging, as the alkaline conditions required for adequate resolution lead to elevated electric current and accelerated capillary deterioration. Analytical parameters, including the nature of the background electrolyte (BGE) and ionic strength, were optimized to ensure reliable separation of phenol, *m*‐cresol, and the internal standard. SALLE was performed using acetonitrile (ACN) as the extraction solvent and ammonium acetate as the salting‐out agent. CZE separation was completed in less than 6 min using a carbonate buffer at pH 10.3. The SALLE procedure yielded good recoveries (96%–102%) and repeatability (relative standard deviation [RSD] for concentration determination < 5.5%). The SALLE–CZE–UV methodology showed excellent linearity (*r^2^* > 0.994) and repeatability (RSD for corrected migration times: 0.4% for phenol and 0.5% for *m*‐cresol; RSD for the ratio of corrected peak areas [analyte/internal standard]: 2.6% for phenol and 1.8% for *m*‐cresol). Moreover, appropriate limits of detection (LOD) and quantification (LOQ) were obtained for insulin formulations in line with regulatory requirements. In addition, suitable LOD and LOQ (0.014; 0.047 g.L^−1^ for phenol and 0.007; 0.022 g.L^−1^ for *m*‐cresol) were obtained for spiked human plasma.

## Graphical abstract

A rapid and reliable SALLE–CZE–UV method was developed for the determination of phenolic preservatives in insulin formulations and human plasma. The approach provides efficient extraction, fast separation (< 6 min), and excellent sensitivity, precision, and recovery, meeting regulatory requirements for accurate quantification.

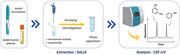

## 1 Introduction

Phenolic preservatives, such as phenol and meta‐cresol, are commonly used in biopharmaceutical formulations, particularly in multidose insulin products intended for repeated use by patients [1]. These compounds inhibit the growth of a broad spectrum of microorganisms except spores during storage and administration [2]. Due to their direct interaction with proteins and their role in maintaining the stability of insulin hexamers, they are considered as critical excipients [3, 4, 5]. However, phenolic preservatives are prone to depletion through degradation or adsorption onto the container surface during insulin storage, especially after dispensing, when recommended storage conditions are often not strictly followed. Typically, insulin prefilled pens and pen cartridges are stored in household refrigerators, which are subject to temperature fluctuations. When used, they are kept at room temperature, which can exceed the recommended temperature (below 30°C), for 2–5 weeks [3, 6]. Thus, unfavorable storage conditions can affect insulin stability and solubility. Furthermore, long term exposure to phenolic preservatives can trigger common side effects of insulin therapy, such as skin irritation, inflammation, and infections, as well as rare but serious side effects such as organ damages due to their cytotoxic effects, inflammatory and hypersensitivity reactions (erythema and anaphylaxis), and genotoxicity [2, 7, 8, 9, 10, 11].

Determining the concentration of phenolic preservatives in insulin formulations and human plasma is highly valuable in ensuring regulatory compliance in quality control and in guaranteeing the efficacy and safety of diabetes treatment. Both insulin formulations and biological matrices (such as human plasma or serum) are complex samples containing salts, organic molecules, and proteins, all of which can interfere with analyses. As a result, analyses are typically preceded by a sample preparation step for extraction and pre‐concentration of targeted analytes. Traditionally, solid phase extraction (SPE) and liquid–liquid extraction (LLE) are employed for the extraction of organic molecules [12, 13]. SPE is costly and involves complex procedures, while LLE is solvent consuming and lacks selectivity. Moreover, many solvents commonly used in LLE are incompatible with advanced analytical techniques such as LC–MS and CE–MS. It requires evaporation and reconstitution in other solvents before analysis due to their water immiscibility and ion suppression effects, which limits their applicability. Furthermore, recent sample preparation techniques such as microextraction by packed sorbent, monolith spin column extraction, microwave‐assisted extraction, and membrane‐assisted solvent extraction, vary in complexity and cost; some require specialized equipment or procedures, while others, like dispersive liquid–liquid microextraction, are generally simple and low‐cost but are not suitable for complex matrices such as plasma or pharmaceutical formulations [14]. Recently, salting‐out assisted liquid–liquid extraction (SALLE) has proven to be more effective than conventional methods for pharmaceutical analysis. It is simple, compatible with most analytical techniques and can be applied to a broad range of molecules [15, 16]. Moreover, SALLE technique is attracting considerable attention for the extraction of chemicals from biological samples due to its efficiency in protein precipitation [17, 18]. However, this technique has not yet been applied to the quantification of phenolic compounds in insulin formulations and human plasma.

In parallel, chromatographic, electrochemical, and spectroscopic methods are commonly used to determine phenolic preservatives in pharmaceutical formulations and biological matrices [3, 19, 20, 21]. However, chromatographic techniques often require time‐consuming sample preparation, expensive instrumentation, and large volumes of organic solvents, which are environmentally burdensome and less suitable for high‐throughput analysis [22]. Mass spectrometry (MS), although highly sensitive and specific, is costly and requires sophisticated equipment and highly trained personnel. Additionally, its compatibility with certain separation methods may be limited depending on the sample matrix and the need for extensive pretreatment [23]. Electrochemical methods, while relatively simple and cost‐effective, can suffer from limited selectivity in complex biological matrices and often require extensive electrode surface modifications to improve sensitivity and reproducibility [24]. These limitations highlight the ongoing need for analytical approaches that combine efficient sample preparation with sensitive, selective, and cost‐effective detection techniques.

In this context, capillary electrophoresis (CE) emerges as a compelling alternative that addresses many of the above limitations. To date, only one method has been developed using CE for phenol and *m*‐cresol determination in pharmaceutical formulations. Indeed, Jaworska et al. [25] proposed a fast micellar electrokinetic chromatography (MEKC) method for the determination of phenol and *m*‐cresol and other preservatives in pharmaceutical formulations. However, MEKC is complex and requires the use of surfactants which limit its sensitivity and compatibility with other techniques such as MS. Despite the potential of capillary zone electrophoresis (CZE) in terms of peak efficiency and resolution [26, 27], it remains underutilized for the determination of phenol and *m*‐cresol with no study reported to date, largely due to the relatively high *p* *K*_a_ values (around 10.0) [28]. These compounds are weak acids that require alkaline conditions to be fully ionized which is necessary to achieve sufficient electrophoretic mobility. However, operating under basic conditions can be challenging due to increased current and potential capillary degradation [29], which may alter separation performance and limit the routine use of CZE for phenolic preservatives analysis. Moreover, the use of background electrolytes (BGEs) with high salt content can negatively affect the performance of SALLE–CZE–UV due to phase separation phenomena, as demonstrated in previous studies [30].

The aim of this study was to develop a single generic analytical methodology that would allow highly efficient separation and accurate quantification of phenol and *m*‐cresol in various insulin formulations and plasma samples. This work proposes to combine SALLE extraction of phenolic preservatives with CZE analysis for the first time. Key parameters of the SALLE procedure, including salt type, concentration, and solvent volume, were optimized using standard solutions. Finally, the performance of the SALLE procedure and CZE analysis were evaluated. The entire methodology was applied for the qualitative analysis of insulin formulations as well as quantitative analysis of phenolic preservatives in insulin formulations and spiked human plasma samples.

## 2 Materials and Methods

### 2.1 Chemicals and Reagents

Sodium hydroxide (NaOH) (≥ 98%), hydrochloric acid (HCl) (≥ 37%), meta‐cresol (99%), phenol (≥ 99%), methylparaben (≥ 99%), dibasic sodium phosphate (≥ 99%), potassium carbonate (≥ 99%), sodium chloride (≥ 99%), magnesium sulphate (≥ 99%), and ACN (≥ 99%) were purchased from Sigma–Aldrich (Germany). Ammonium acetate (≥ 98%) was purchased from Carlo Erba (France). Glacial acetic acid was purchased from Prolabo (France). All reagents were of analytical grade.

Pharmaceutical formulations of insulin glargine (Lantus SoloStar), insulin detemir (Levemir), human insulin (Actrapid), and insulin aspart (Novomix) were provided by several officinal pharmacies.

### 2.2 Solutions, BGEs, and Samples

A standard stock solution of 10 g.L^−1^ of *m*‐cresol was prepared in ACN. Individual stock standard solutions of the analytes (*m*‐cresol and phenol) and internal standard (IS) (methylparaben) were prepared in water at a concentration of 1 g.L^−1^. The working standard solutions of *m*‐cresol, phenol, and methylparaben at the desired concentrations were prepared by mixing the appropriate amounts of the individual standard solutions and diluting them with water.

Stock solutions of NaOH (1 M), HCl (6 M), and the salts dibasic sodium phosphate (0.25 M), potassium carbonate (2 M), ammonium acetate, sodium chloride (2 M), and magnesium sulphate were prepared in water.

The following solutions were used as BGEs: (i) phosphate buffer at pH 12.3 ± 0.1 and various ionic strengths (I) (25, 50, and 75 mM) and (ii) carbonate buffer at pH 10.3 ± 0.1 and various ionic strengths (50, 100, and 150 mM). The pH of phosphate buffer was adjusted using NaOH (1 M) and the pH of carbonate buffer was adjusted using HCl (6 M). The exact pH value of each BGE was measured with a potentiometer (Mettler Toledo, France). All solutions were kept at 4°C –8°C.

### 2.3 Optimized SALLE Procedure

The SALLE procedure was applied to individual or mixed aqueous standard solutions of *m*‐cresol and phenol, spiked plasma samples with *m*‐cresol and phenol, and spiked samples of insulin formulations with *m*‐cresol or *m*‐cresol and phenol. Appropriate volumes of samples (10 µL of insulin formulation or 60 µL of human plasma) were transferred into a conical tube, followed by the addition of a fixed amount of IS (60 µL of methylparaben, 1 g·L^−1^). The samples were then spiked with appropriate volumes of *m*‐cresol alone or *m*‐cresol and phenol stock solutions, depending on the desired concentration. Water was added to bring the final volume to 300 µL. A 300 µL solution of ammonium acetate (2 M) was added and followed by 600 µL of ACN to obtain a 50/50 (v/v) sample/ACN ratio. The mixture was vortexed for 1 min and centrifuged for 3 min (5000 rpm, 20°C). Two phases were obtained: a lower aqueous phase and an upper organic phase. Blank solutions were prepared using the same procedure.

### 2.4 Extraction Recovery Determination

The extraction recovery was determined by comparing the concentration of extracted preservatives from aqueous standard solutions at three concentration levels (0.05, 0.10, and 0.15 g.L^−1^) and corresponding post‐extraction (organic phase) spiked standards with phenol, *m*‐cresol, and IS for 3 days. Post‐extraction spiked standards were prepared using the organic phase obtained from SALLE extraction of a blank (water) that was spiked with known amounts of phenol and *m*‐cresol.

The extraction recovery percentage was calculated using Equation (1):

(1) $$\begin{array}{c} \mathrm{Extraction}\;\mathrm{recovery}=\frac{\mathrm{mean}\;C\;\mathrm{of}\;\mathrm{extracted}\;\mathrm{standard}}{\mathrm{mean}\;C\;\mathrm{of}\;\mathrm{post}-\;\mathrm{extraction}\;\mathrm{spiked}\;\mathrm{standard}}\;.100 \end{array}$$

Calibration curves of $\frac{\mathrm{CPA}\;\mathrm{of}\;\mathrm{analyte}}{\mathrm{CPA}\;\mathrm{of}\;\mathrm{internal}\;\mathrm{standard}}$ against $\frac{C\;\mathrm{of}\;\mathrm{analyte}}{C\;\mathrm{of}\;\mathrm{internal}\;\mathrm{standard}}$ were constructed each day to calculate the concentration of analytes in extracted samples, where *C* is the concentration of considered analyte (g.L^−1^) and CPA is the corrected peak area of the considered analyte in the electropherogram that is equal to the peak area of the considered analyte divided by its migration time.

### 2.5 Application

Standard solutions were prepared in diverse matrices: water, plasma, and insulin formulations.

The calibration curves of $\frac{\mathrm{CPA}\;\mathrm{of}\;\mathrm{analyte}}{\mathrm{CPA}\;\mathrm{of}\;\mathrm{internal}\;\mathrm{standard}}$ against $\frac{C\;\mathrm{of}\;\mathrm{analyte}}{C\;\mathrm{of}\;\mathrm{internal}\;\mathrm{standard}}$ were constructed to perform quantification of phenol and/or *m*‐cresol using internal calibration method for water and plasma samples, and standard addition method for insulin formulations.

### 2.6 Instrumentation and Operating Conditions

The CE–UV analyses were performed using a Sciex PA800 CE system (Beckman, USA) equipped with a UV detector. Polymicro fused silica capillaries (Molex, USA) (id = 50 µm, L_eff_ = 20 cm, and L = 30 cm) were used for electrophoretic separations. The autosampler and capillary cartridge temperatures were set to 8°C and 25°C, respectively. The pre‐conditioning of the capillary was performed by successive rinses with 1 M NaOH and water by applying a pressure of 20 psi for 10 min. Prior to each run, the capillary was rinsed successively with 1 M NaOH, water, and BGE by applying a pressure of 20 psi for 5 min. Samples were injected hydrodynamically by applying a pressure of 0.5 psi for 5–20 s (corresponding to a sample volume from 9.9 to 39.6 nL). Separations were achieved at 10 kV and UV detection was performed at 254 nm. Data were acquired using 32 Karat software (version 8.0, Beckman Coulter).

### 2.7 Limits of Detection (LOD) and Limits of Quantification (LOQ) Calculation

The LOD and LOQ were calculated following the ICH Q2 guidelines [31]:

(2) $$\mathrm{LOD}=3,3\;\;\frac{\sigma }{S}$$

and

(3) $$\mathrm{LOQ}=10\;\;\frac{\sigma }{S}$$

where σ is the residual standard deviation of regression line and S is the slope of the calibration curve.

## 3 Results and Discussion

The study aim was to develop a versatile and robust analytical method for the analysis of phenol and *m*‐cresol in various formulations and plasma samples. The simplest possible electrophoretic analysis conditions were therefore at the heart of our developments. Basic BGEs (pH > 10.0) were therefore selected, under which the target analytes are negatively charged and exhibit intrinsic electrophoretic mobility. Accordingly, phosphate (pH 12.3) and carbonate (pH 10.3) buffers were initially evaluated as BGEs. Alternative buffers such as CHES and CAPS were not investigated because of their UV absorption and cost. Ammonia was not investigated due to its lower buffering capacity at pH > 10.0, safety, and odor issues.

**FIGURE 1:**
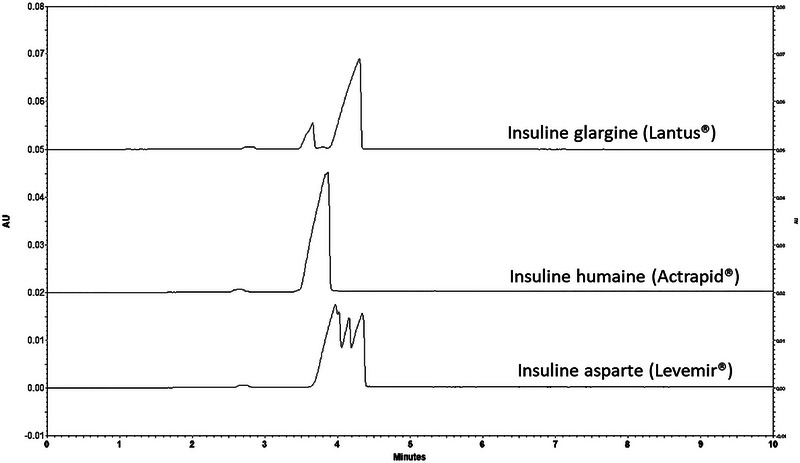
Analysis of diluted insulin formulations (1/10) with phosphate buffer (pH 12.3, I 50 mM) (BGE 2). Experimental conditions: silica capillary (id = 50 µm, L = 30 cm, and L_eff_ = 20 cm), injection: 0.5 psi for 20 s, temperature: 25°C, separation voltage: 10 kV, and detection: 254 nm.

Preliminary experiments involving the direct determination of phenolic preservatives using phosphate and carbonate BGEs demonstrated that dilution alone was insufficient. Even after extensive dilution, peak overlap between insulin and phenol/*m*‐cresol persisted (Figure 1). In addition, electrophoretic profiles varied significantly among different insulin formulations. As a result, a simple dilute‐and‐shoot approach could not provide reliable separation across multiple insulin formulations or in human plasma. Indeed, direct injection presents several challenges: (i) Direct determination of phenolic preservatives lacks versatility, as separation conditions must be optimized individually for each insulin formulation due to differences in the physicochemical properties of insulin analogs and formulation heterogeneity. (ii) At pH values above 10, phenolic preservatives are predominantly charged, but insulin and other proteins may suffer from stability issues, leading to the formation of interfering degradation products. (iii) Under acidic pH conditions, insulins are positively charged, requiring positively charged or neutral capillary coatings, while phenolic preservatives remain neutral and cannot be separated electrophoretically. Hence, it is necessary to extract the phenolic preservatives or modify their properties by complexation with charged molecules or derivatization to allow their analysis by CZE. In the present case study, the SALLE approach was applied to remove matrix interferences (proteins, organic molecules, and ions) of both plasma and insulin formulations and ensure robust CZE analysis.

### 3.1 Optimization of the Phenolic Preservatives Separation

The two main phenolic preservatives, *m*‐cresol and phenol, which are used in insulin formulations were selected for this study (Table 1). These compounds possess relatively high *p* *K*_a_ values, both around 10.0 [28], implying that their ionization, and consequently their electrophoretic mobility, is strongly dependent on the pH of the BGE. Therefore, basic conditions are required in CZE to ensure that these weak acids are sufficiently ionized for effective migration and resolution. However, performing CZE under strongly alkaline conditions presents several analytical challenges. First, under strongly basic conditions, some buffers exhibit high conductivity, thereby raising the current and potentially causing excessive Joule heating [29]. This not only compromises the efficiency and reproducibility of the separation but may also cause thermal degradation of the external polyimide coating and reduce capillary lifespan. Additionally, maintaining the chemical stability of both the capillary and the analytes requires careful optimization of pH and ionic strength to balance sufficient ionization with overall system robustness, particularly under strongly basic conditions.

**TABLE 1:** Structure and physicochemical properties of phenol, *m*‐cresol, and methylparaben (pubchem.ncbi.nlm.nih.gov/).

Compounds	Phenol	Meta‐cresol	Methylparaben
Structure	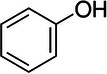	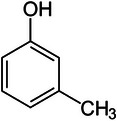	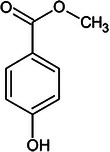
Molecular weight (g.mol^−1^)	94.11	108.14	152.15
Log *P* (o/w)	1.46	1.96	1.96
*p* *K*_a_	10.09	9.99	8.5

To address these challenges, the electrophoretic behavior of the IS (methylparaben), *m*‐cresol, and phenol was evaluated using phosphate buffer at pH 12.3 and carbonate buffer at pH 10.3, with varying ionic strengths. These two BGEs were selected based on their buffering capacity in alkaline conditions and their ability to maintain a stable basic pH. A pH higher than *p* *K*_a_ is essential to ensure the deprotonation of phenol and *m*‐cresol, thus converting them into their anionic forms required for efficient electrophoretic migration. Phosphate and carbonate buffers are commonly used in CZE due to their well‐characterized electrochemical behavior, compatibility with UV detection, and relatively low UV absorbance. Moreover, these buffers offer different ionic mobilities and buffering strengths, which allowed the comparison of separation performance, migration time, and current stability under various analytical conditions. Methylparaben was selected as the IS because of its electrophoretic behavior similar to that of the target phenolic compounds, thereby ensuring high precision and accuracy. It was added at the beginning of the sample preparation procedure and served to compensate for potential analyte losses during extraction as well as for variability in injection and CE migration.

**FIGURE 2:**
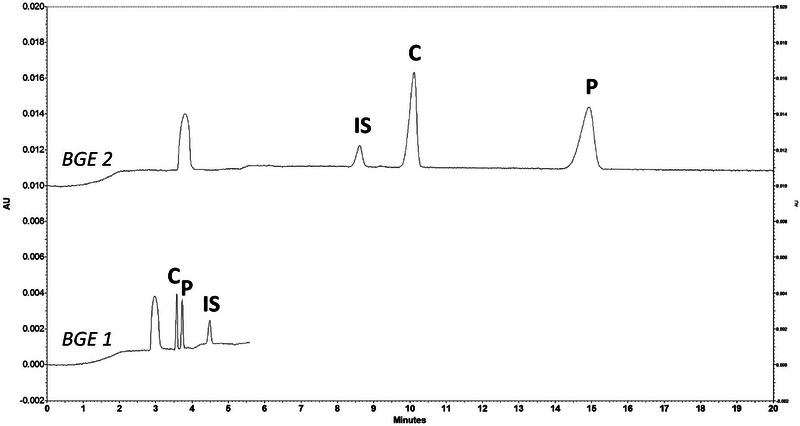
Separation of phenolic preservatives with carbonate buffer (pH 10.3, I 150 mM) (BGE 1) and phosphate buffer (pH 12.3, I 50 mM) (BGE 2). Standard aqueous solution of phenol (P) (0.04 g.L^−1^), *m*‐cresol (C) (0.05 g.L^−1^), and internal standard (IS) (0.04 g.L^−1^). Experimental conditions: silica capillary (id = 50 µm, L = 30 cm, and L_eff_ = 20 cm), injection: 0.5 psi for 20 s, temperature: 25°C, separation voltage: 10 kV, and detection: 254 nm.

Excellent separation of all three compounds was achieved under optimal ionic strengths: 50 mM for phosphate and 150 mM for carbonate. Under these conditions, stable electric currents (approximately 80 µA for phosphate and 100 µA for carbonate at low injection volumes) were maintained, minimizing Joule heating and preventing capillary damage. Shorter analysis times were achieved with carbonate buffers compared to phosphate buffers, thereby improving throughput without compromising resolution (Figure 2). The ionization and thus electrophoretic mobilities of *m*‐cresol and phenol being particularly affected between pH 10.3 and 12.3, they migrate before the IS with the carbonate buffer and after with the phosphate buffer. Due to shorter analysis time and satisfactory separation, the carbonate buffer (pH 10.3, I 150 mM) was selected for subsequent experiments.

Taking into account both analysis time and separation quality, carbonate buffer at pH 10.3 with an ionic strength of 150 mM enabled sufficient ionization of phenol and *m*‐cresol (typically > 60% ionized at pH = *p* *K*_a_ + 1), while maintaining shorter migration times and better analytical throughput. Furthermore, the method demonstrated excellent repeatability, with relative standard deviations (RSD) of migration times and peak areas consistently below 2% (*n* = 6, sample containing phenol [0.04 g.L^−1^], *m*‐cresol [0.05 g.L^−1^], and IS [0.04 g.L^−1^]), confirming its robustness for quantitative analyses. In contrast, the phosphate buffer at pH 12.3 ensured complete ionization of the analytes; it resulted in longer migration times and increased system stress. So, carbonate buffer at pH 10.3 was selected as the optimal BGE for subsequent experiments.

### 3.2 Optimization of the SALLE Procedure

Insulin formulations and human plasma are complex samples containing proteins, organic molecules, and electrolytes. Accordingly, SALLE of phenol and *m*‐cresol was conducted to avoid matrix interferences during their quantification as a versatile approach. In SALLE, a water‐miscible solvent and a salt are added to an aqueous sample to form a biphasic system, in which organic compounds are transferred into the organic phase [32]. Therefore, the nature of the extraction solvent and the salting‐out agent are crucial variables to consider.

**FIGURE 3:**
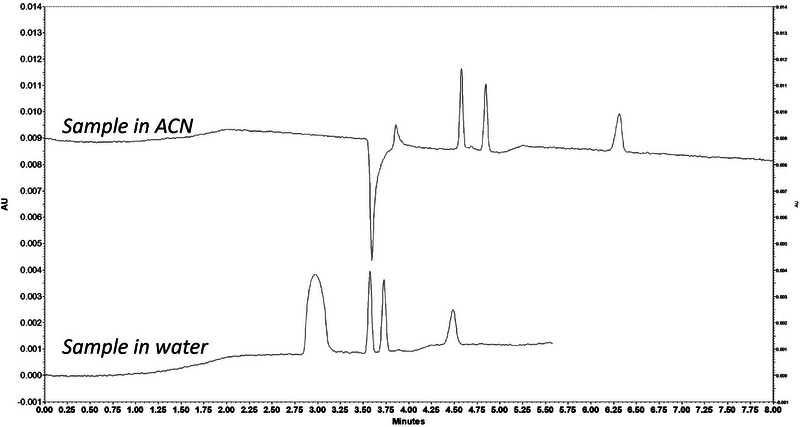
Effect of ACN on electrophoretic profile of phenolic preservatives with internal standard samples. Standard solution of phenol (0.04 g.L^−1^), *m*‐cresol (0.05 g.L^−1^), and IS (0.04 g.L^−1^) in water and ACN. Experimental conditions: silica capillary (id = 50 µm, L = 30 cm, and L_eff_ = 20 cm), BGE: carbonate buffer (pH 10.3, I 150 mM), injection: 0.5 psi for 20 s, temperature: 25°C, separation voltage: 10 kV, and detection: 254 nm.

Among commonly used water‐miscible solvents (alcohols, acetone, tetrahydrofuran, and ACN), ACN was selected based on the following reasons: (i) high solubility of phenolic preservatives in ACN; (ii) low solubility of excipients present in insulin formulations such as polysorbate 20 and glycerol in ACN; (iii) lower water content in ACN compared to alcohols and acetone; (iv) protein precipitation induced by ACN; (v) cleaner extracts compared to other solvents [17, 33]. To evaluate the effect of ACN on the electrophoretic behavior of target analytes, a mixture of *m*‐cresol, phenol, and the IS methylparaben was prepared in ACN and analyzed by CE. The presence of ACN in the sample matrix caused a slight increase in migration times, likely due to changes in sample conductivity and viscosity, but also led to an improvement in separation resolution between the analytes (Figure 3), confirming the suitability of ACN as an extraction solvent in this analytical method.

**FIGURE 4:**
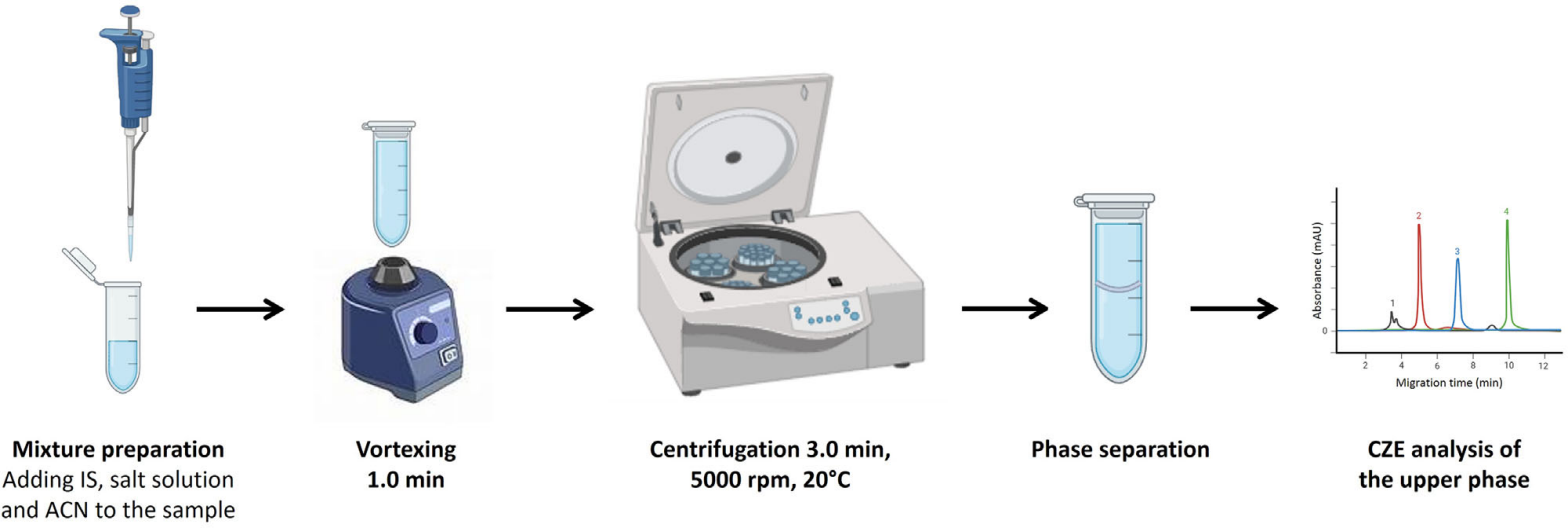
SALLE procedure for analysis of phenolic preservatives.

Figure 4 shows a schematic representation of the steps involved in the SALLE process to achieve separation between the aqueous and organic phases. Briefly, samples were spiked with methylparaben, diluted to a total volume of 300 µL with water, and then 300 µL of salt solution was added, making a total aqueous phase volume of 600 µL. Then, 600 µL of ACN was added. The mixture was vortexed and centrifuged to allow phase separation. The organic phase was collected and analyzed by CE. These conditions were established from preliminary tests that demonstrated phase separation and effective extraction performance.

**FIGURE 5:**
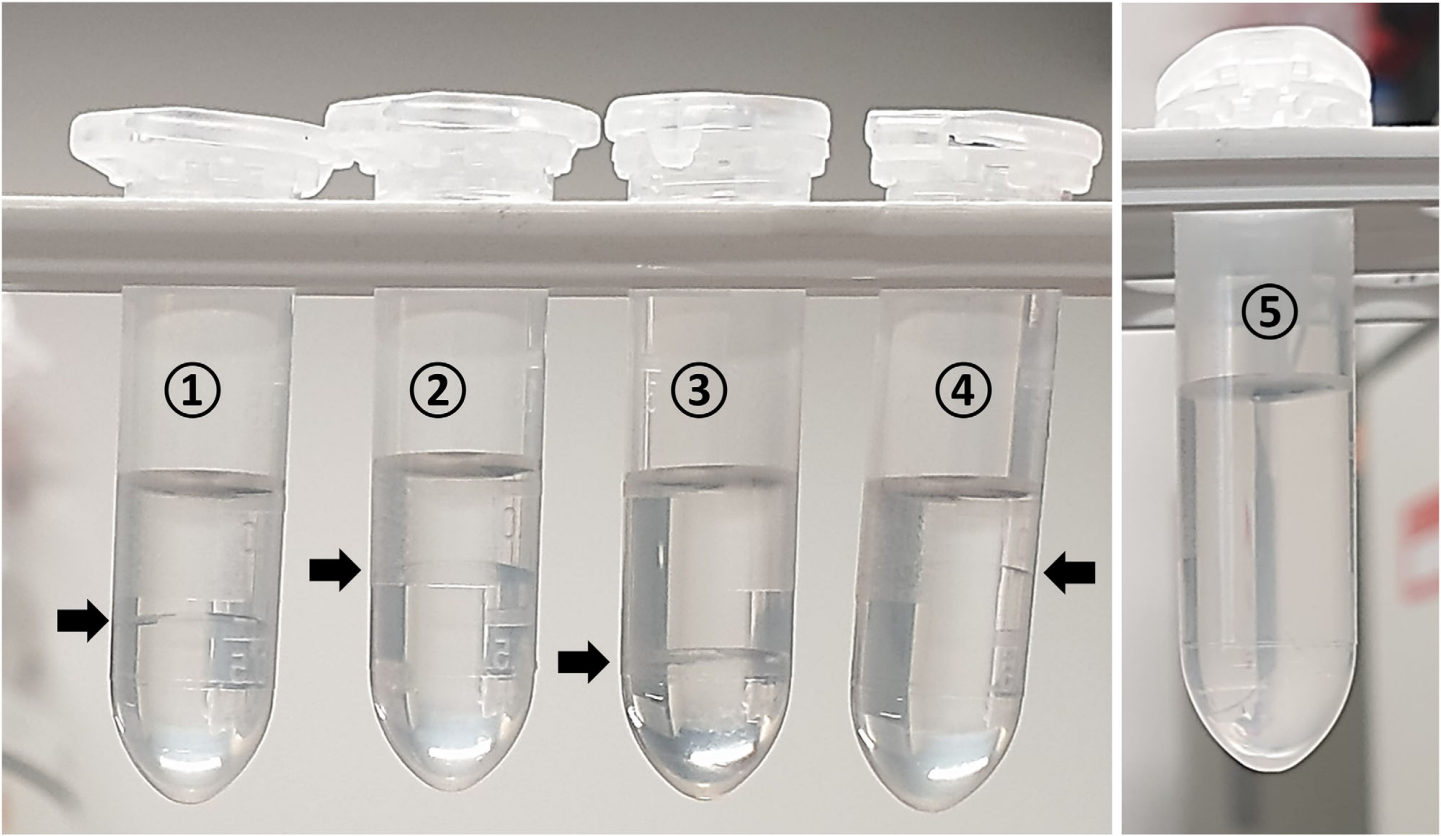
Post‐extraction images of standard solution with different salts: ① potassium carbonate, ② ammonium acetate, ③ sodium chloride, ④ magnesium sulphate, and ⑤ control sample (600 µL of water mixed with 600 µL of ACN). Experimental conditions: 300 µL of salt (2 M), 300 µL standard solution (IS [0.04 g.L^−1^], *m*‐cresol [0.05 g.L^−1^], and phenol [0.04 g.L^−1^]), and 600 µL of ACN.

In order to optimize the SALLE procedure, we investigated how the nature of the salting‐out agent affects phase separation. The following salts were tested: sodium chloride, potassium carbonate, ammonium acetate, and magnesium sulphate at equal concentration. Results are shown in Figure 5. Ammonium acetate produced clear phase separation within a few seconds after shaking (without centrifugation). In contrast, interface formation between the two phases was slow (taking a few minutes without centrifugation) with the other salts. In addition, the volume of the upper phase obtained after SALLE was influenced by the nature of the salt, in the following order: sodium chloride < ammonium acetate < potassium carbonate < magnesium sulphate. This can be explained by the presence of residual salt water residue in the organic phase, which depends on salt concentration, as well as the differences in physicochemical properties of salts (molecular weight, surface tension and ionic strength) and the salting‐out effect following the Hofmeister series of salt anions [16, 34, 35]. The best electrophoretic profiles were obtained with ammonium acetate and sodium chloride. Lower peak heights were obtained with potassium carbonate, and the use of magnesium sulphate caused capillary clogging. Ammonium acetate was selected for further analyses as it provided fast phase separation and good electrophoretic profile. Its concentration was then varied from 0.1 to 2.0 M. Phase separation was achieved only at 2.0 M. Under these conditions, no peak was detected in the aqueous phase, indicating that the analytes were mostly transferred into the organic phase.

**FIGURE 6:**
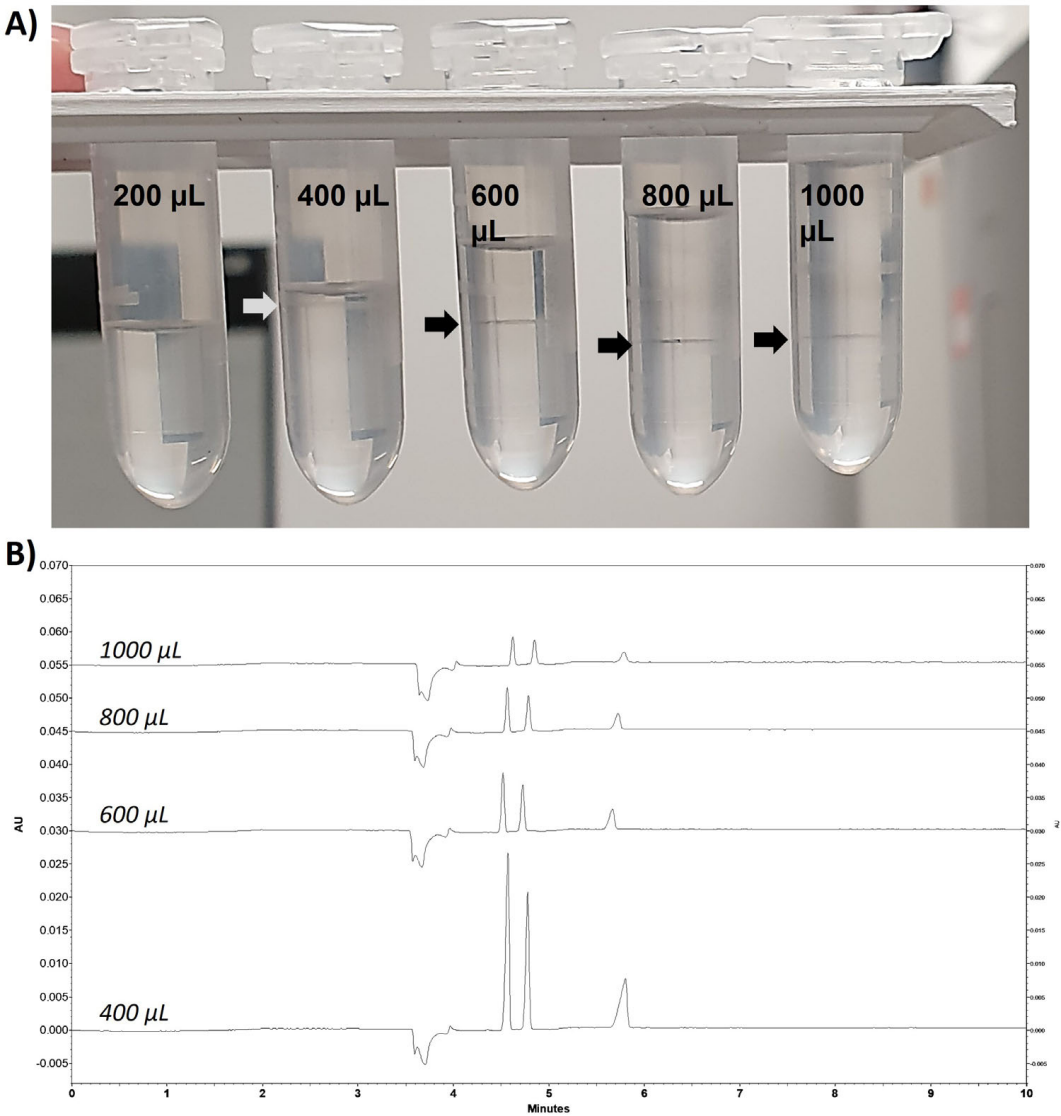
Effect of ACN volume on electrophoretic profile of post‐extraction solution of phenol, *m*‐cresol, and IS. Experimental conditions: (A) SALLE: a 300 µL solution of ammonium acetate (2 M), 300 µL standard solution (IS [0.04 g.L^−1^], *m*‐cresol [0.05 g.L^−1^], and phenol [0.04 g.L^−1^]), and different volumes of ACN (200, 400, 600, 800, and 1000 µL). (B) CE separation: silica capillary (id = 50 µm, L = 30 cm, and L_eff_ = 20 cm), BGE: carbonate buffer (pH 10.3, I 150 mM), injection: 0.5 psi for 20 s, temperature: 25°C, separation voltage: 10 kV, and detection: 254 nm.

Next, the solvent volume was optimized to ensure efficient phase separation, minimize dilution effects, and reduce the volume of ACN, while focusing on effective removal of matrix interferences rather than pre‐concentration of the analytes [36, 37]. To this end, different volumes of ACN (200, 400, 600, 800, and 1000 µL) were tested by adding them to a fixed 600 µL aqueous phase containing the analytes, IS, and salt solution (Figure 6). When 200 µL of ACN was added, phase separation was incomplete, resulting in poor extraction efficiency. Increasing the ACN volume to 400 µL improved the extraction efficiency, as evidenced by higher peak intensities in the electropherograms. However, the resulting organic phase volume was relatively small, which complicated the sampling process and could increase variability. At 600 µL of ACN, a clear biphasic system was achieved with sufficient organic phase volume, allowing both effective analyte enrichment and ease of sampling. Higher volumes of ACN (800 and 1000 µL) further increased the organic phase volume but did not significantly enhance analyte recovery, and would lead to unnecessary solvent consumption. Therefore, 600 µL of ACN was selected as the optimal volume, providing a practical compromise between sensitivity, phase separation efficiency, and operational convenience.

### 3.3 Evaluation of the Methodology Performance

Under optimized conditions, a comprehensive extraction recovery study was conducted to evaluate the efficiency and reliability of the SALLE procedure for isolating *m*‐cresol, phenol, and the IS from standard aqueous solutions. The study was performed at three distinct concentration levels, low (0.05 g·L^−1^), medium (0.10 g·L^−1^), and high (0.15 g·L^−1^) of each analyte to cover the typical analytical range expected for these analytes in real samples (Table 2). The three samples were prepared by conducting the SALLE extraction of standard aqueous solutions containing *m*‐cresol, phenol, and fixed concentration of IS (0.1 g·L^−1^). Each concentration level was tested in triplicate over a period of 3 consecutive days to assess the reproducibility and stability of the extraction method over time. To quantify the extracted analytes at the three levels of concentration, post‐extraction spiked standards were prepared by spiking the organic phase obtained from SALLE extraction of a blank with known amounts of phenol, *m*‐cresol, and the IS. These standards were then used to generate calibration curves. This approach allowed to consider the losses during extraction and matrix effect.

**TABLE 2:** Results of SALLE extraction recovery of phenolic preservatives.

Concentration level (g.L^−1^)	Mean found concentrations (g.L^−1^) *n* = 3		RSD (%) *n* = 3		Recovery (%)	
	Phenol	*m*‐cresol	Phenol	*m*‐cresol	Phenol	*m*‐cresol
0.050	0.05	0.05	3.27	5.48	99.56	100.72
0.100	0.10	0.10	1.10	2.51	100.11	101.96
0.150	0.15	0.15	1.51	2.05	99.73	99.68

These calibration curves were constructed using six concentration points ranging from 0.025 to 0.150 g·L^−1^. Excellent linearity of the calibration curves was evidenced by mean coefficients of determination (*r^2^*) of 0.991 for phenol and 0.998 for *m*‐cresol. These curves served as references to calculate the concentrations of analytes in the extracted samples.

Extraction recoveries were calculated by taking the ratio of the mean concentration of the analytes obtained after extraction to the mean concentration of post‐extraction spiked standards, thereby providing a direct measure of the method's extraction efficiency. The results demonstrated excellent recoveries for both phenol and *m*‐cresol, with values consistently falling between 99.6% and 102% (Table 2). Such high recovery percentages indicate that the SALLE procedure effectively extracts the analytes from the aqueous solutions with minimal loss, validating its suitability for quantitative analysis. Furthermore, the repeatability of the method was assessed by calculating the RSD of the determined concentrations (0.05, 0.10, and 0.15 g.L^−1^) across extraction replicates (*n* = 3). The RSD values were found to be below 3.3% for phenol and below 5.5% for *m*‐cresol, underscoring the repeatability and robustness of the SALLE extraction process.

In addition to the extraction step, the repeatability of the SALLE–CZE–UV analysis was evaluated. Six independent extracts of a standard solution at a concentration of 0.150 g·L^−1^, were analyzed to determine the consistency of the electrophoretic separation and quantification. The RSD for the corrected migration times was low, at 0.4% for phenol and 0.5% for *m*‐cresol, demonstrating highly stable and reproducible migration behavior during the electrophoretic runs. Likewise, the RSD values for the ratio of the CPA of the analyte to the CPA of the IS (*n* = 3) were 2.6% for phenol and 1.8% for *m*‐cresol, indicating consistent detector response and quantification accuracy.

These results confirm that the SALLE–CZE–UV methodology offers excellent performance characteristics, including high extraction recovery, strong repeatability, and precise quantification. Consequently, this approach can be confidently applied for the quantitative analysis of phenolic preservatives in complex matrices such as pharmaceutical formulations and biological samples.

### 3.4 Application

**FIGURE 7:**
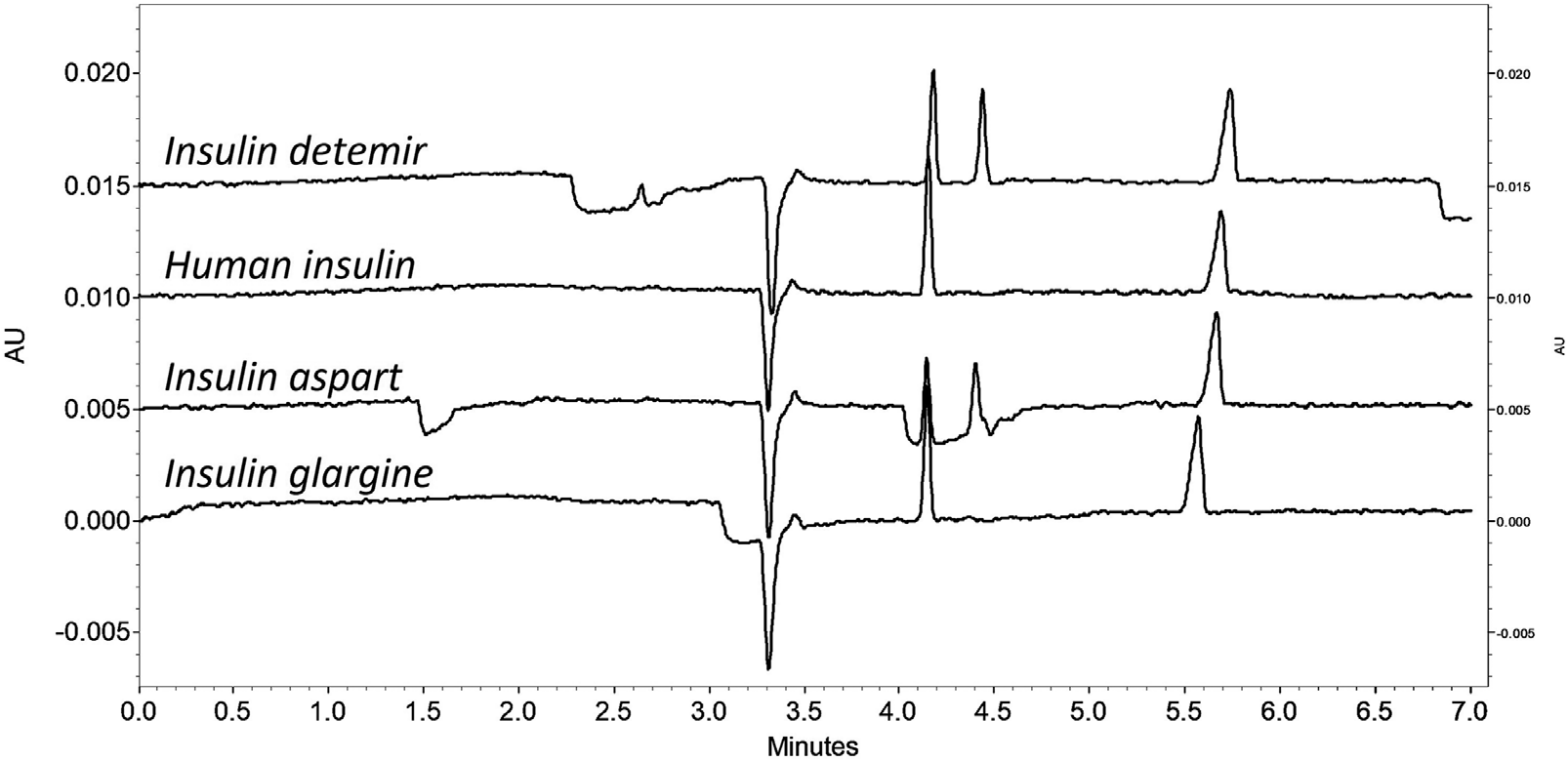
Electropherograms of phenolic preservatives extracted from insulin formulations spiked with IS. Experimental conditions: silica capillary (id = 50 µm, L = 30 cm, and L_eff_ = 20 cm), BGE: carbonate buffer (pH 10.3, I 150 mM), injection: 0.5 psi for 5 s, temperature: 25°C, separation voltage: 10 kV, and detection: 254 nm.

The SALLE–CZE–UV methodology was applied to extract phenolic preservatives from four commercial insulin formulations including human insulin, its rapid acting analog (insulin aspart), and its long‐acting analogs (insulin glargine and insulin detemir). Figure 7 shows the electropherograms obtained for the extracted phenolic preservatives under optimized conditions in the presence of the IS. It indicates the presence of *m*‐cresol in all insulin formulations and of phenol in the insulin aspart and insulin detemir formulations. These findings are in agreement with the corresponding summaries of product characteristics available from the European Medicines Agency (https://www.ema.europa.eu/), which report the inclusion of both phenol and *m*‐cresol in insulin aspart and insulin detemir, and *m*‐cresol alone in human insulin and insulin glargine. However, it is important to note that these regulatory documents do not specify the exact concentrations of the preservatives.

**FIGURE 8:**
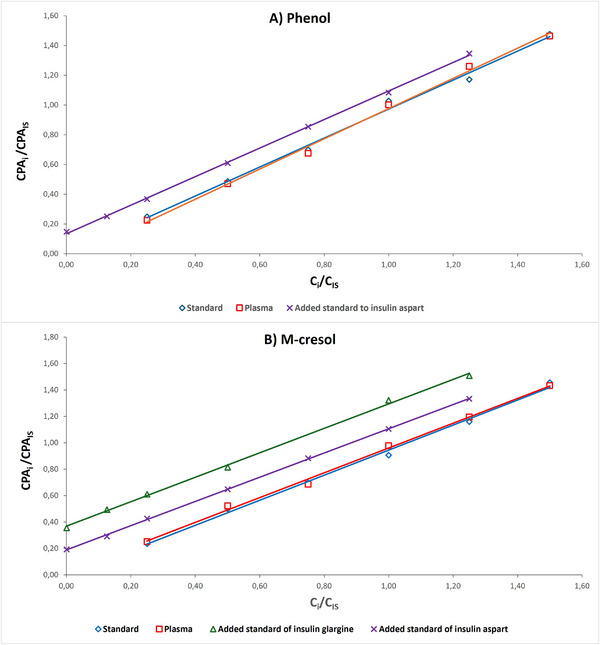
Calibration curves obtained by internal calibration of phenolic preservatives in water and plasma, and standard addition calibration of phenolic preservatives in insulin formulations with (A) phenol and (B) *m*‐cresol. Experimental conditions: silica capillary (id = 50 µm, L_T_ = 30 cm, and L_eff_ = 20 cm), IS concentration: 0.1 g.L^−1^, BGE: carbonate buffer (pH 10.3, I 150 mM), injection: 0.5 psi for 5 s, temperature: 25°C, separation voltage: 10 kV, and detection: 254 nm.

The SALLE–CZE–UV methodology was then applied for the analysis of phenolic preservatives in human plasma spiked samples and two insulin formulations (insulin aspart and insulin glargine). IS calibration was applied for human plasma spiked standards and addition standard calibration was applied for insulin formulations to address the matrix effects as the exact composition of the formulations is not available (Table 3). The calibration standard solutions with IS were used to determine linearity and linear ranges of the CZE methods. The coefficients of determination were from 0.994 to 0.999 and the linear ranges were between 0.025 and 0.150 g.L^−1^ for human plasma spiked standards. A statistical comparison using *t*‐test of the calibration curves of the extracts obtained from standard solutions in water and from spiked human plasma (Figure 8) revealed no significant difference between the two slopes (*p* > 0.05 for both slopes and intercepts), which confirms the specificity of the method and the absence of matrix effect. Similarly, slopes of standard addition calibration curves for the two insulin formulations were found to be statistically identical to those for water and spiked plasma, demonstrating again the specificity of the methodology. Differences in intercepts are solely due to the initial presence of phenol and/or cresol in the formulations.

**TABLE 3:** Summary of calibration curves parameters of phenolic preservatives.

Analyte	Parameter	Water	Plasma	Insulin aspart	Insulin glargine
Phenol	Linear range (g.L^−1^)	0.025–0.150	0.025–0.150	0.026–0.139	
	Slope ± SD	0.975 ± 0.038	1.016 ± 0.031	0.961 ± 0.009	
	Intercept ± SD	−0.002 ± 0.037	−0.040 ± 0.030	0.134 ± 0.007	
	*r^2^*	0.9940	0.9924	0.9995	
	LOD; LOQ (g.L^−1^)	0.013; 0.041	0.015; 0.047	0.004; 0.011	
*m*‐cresol	Linear range (g.L^−1^)	0.025–0.150	0.025–0.150	0.034–0.146	0.052–0.165
	Slope ± SD	0.948 ± 0.031	0.939 ± 0.026	0.918 ± 0.007	0.927 ± 0.019
	Intercept ± SD	−0.003 ± 0.030	0.022 ± 0.025	0.189 ± 0.005	0.368 ± 0.013
	*r^2^*	0.9957	0.9970	0.9998	0.9983
	LOD; LOQ (g.L^−1^)	0.011; 0.034	0.009; 0.029	0.003; 0.008	0.007; 0.023

Abbreviation : SD, standard deviation.

The determination of phenolic preservatives in insulin formulations yielded the following values: 0.93 g.L^−1^ of phenol and 1.25 g.L^−1^ of *m*‐cresol in insulin aspart, and 2.4 g.L^−1^ of *m*‐cresol in insulin glargine. These values are within the usual range of 0.07%–5% (w/v) for phenol and 0.17%–0.3% (w/v) for meta‐cresol incorporated in insulin formulations [38].

## 4 Concluding Remarks

A simple, fast, inexpensive, and reliable analytical methodology is proposed for qualitative and quantitative analyses of phenolic preservatives in commercial insulin formulations and human plasma samples. The methodology is performed in two steps: (i) sample preparation using SALLE with excellent extraction yield and (ii) high‐speed separation resolution CZE analysis under alkaline conditions, necessary to fully ionize the weakly acidic phenolic compounds and ensure adequate electrophoretic mobility. The SALLE extraction and CZE separation procedures for phenol, *m*‐cresol, and methylparaben have been optimized. The samples were mixed with appropriate volumes of water, ammonium acetate (2 M), and ACN to get 50/50 (v/v) water/ACN, and centrifugated. The organic phase was then collected and analyzed by CZE using a carbonate buffer (pH 10.3, I 150 mM) as BGE. This methodology demonstrates good performance in terms of repeatability of sample preparation (extraction recovery above 99.6%), CZE separation (RSD on determined concentrations below 5.5%), and linearity (*r^2^* > 0.99). In addition, it enables successful qualitative and quantitative analyses of phenolic preservatives in human plasma spiked samples and human insulin formulations and its rapid‐acting and long‐acting analogs with adequate LOD and LOQ. Further studies will focus on methodology validation, sample preparation automation, and expanding the application field of the methodology.

## Ethics Statement

The work presented in this manuscript was conducted in strict accordance with applicable ethical principles. All experimental procedures complied with relevant national and international regulations, as well as institutional guidelines.

## Conflicts of Interest

The authors declare no conflicts of interest.

## Data Availability

The data that support the findings of this study are available from the corresponding author upon reasonable request.
